# Cold Tumors: A Therapeutic Challenge for Immunotherapy

**DOI:** 10.3389/fimmu.2019.00168

**Published:** 2019-02-08

**Authors:** Paola Bonaventura, Tala Shekarian, Vincent Alcazer, Jenny Valladeau-Guilemond, Sandrine Valsesia-Wittmann, Sebastian Amigorena, Christophe Caux, Stéphane Depil

**Affiliations:** ^1^Centre Léon Bérard, Lyon, France; ^2^INSERM U1052, Centre de Recherche en Cancérologie de Lyon, Lyon, France; ^3^Institut Curie, PSL Research University, INSERM, U932, Paris, France; ^4^Université Claude Bernard Lyon 1, Lyon, France

**Keywords:** cold tumors, T cells, tumor antigen, presentation, priming, trafficking, immunotherapy

## Abstract

Therapeutic monoclonal antibodies targeting immune checkpoints (ICPs) have changed the treatment landscape of many tumors. However, response rate remains relatively low in most cases. A major factor involved in initial resistance to ICP inhibitors is the lack or paucity of tumor T cell infiltration, characterizing the so-called “cold tumors.” In this review, we describe the main mechanisms involved in the absence of T cell infiltration, including lack of tumor antigens, defect in antigen presentation, absence of T cell activation and deficit of homing into the tumor bed. We discuss then the different therapeutic approaches that could turn cold into hot tumors. In this way, specific therapies are proposed according to their mechanism of action. In addition, ‘‘supra-physiological’’ therapies, such as T cell recruiting bispecific antibodies and Chimeric Antigen Receptor (CAR) T cells, may be active regardless of the mechanism involved, especially in MHC class I negative tumors. The determination of the main factors implicated in the lack of preexisting tumor T cell infiltration is crucial for the development of adapted algorithms of treatments for cold tumors.

Immune checkpoint inhibitors (ICIs) have changed the treatment landscape of many tumors, inducing durable responses in some cases, Tumor mutational load, CD8^+^ T cell density and Programmed cell Death Ligand−1 (PD-L1) expression have each been proposed as distinct biomarkers of response to PD-1/-L1 antagonists. The lymphocyte infiltration and IFN-γ status may be key factors for effective anti-PD-1/-L1 therapy by defining a “T cell inflamed” phenotype (“hot tumors”). In contrast, lack of T cells infiltrating the tumor characterizes “non-inflamed” or “cold tumors” (in which other immune populations or myeloid cells can however be observed). Immunological treatment of cold tumors is a great challenge as no adaptive immune response has been set up or maintained. In this review, we discuss the possible issues that the immune system could encounter at different steps of the anti-tumor immune cycle (1), leading to the absence of T cell infiltration: lack of tumor antigens, defect in Antigen Presenting Cells (APCs), absence of T cell activation and deficit of homing into the tumor bed (Figure 1). The potential therapeutic strategies to overcome these problems will be described in the second part of this review. We will not discuss here the mechanisms of immune escape developed by inflamed tumors, reviewed elsewhere (2).

**Figure 1:**
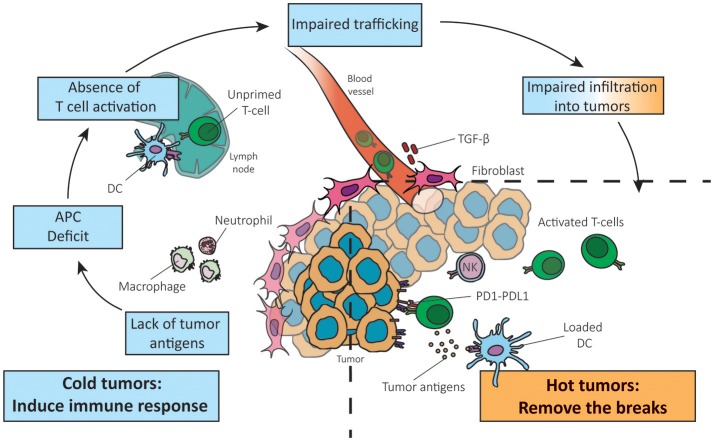
Reversing a cold into a hot tumor. Adapted from Chen and Mellman (1). The absence of T cells in the tumor can be due to the lack of tumor antigens, APC deficit, absence of T cell priming/activation and impaired trafficking of T cells to the tumor mass (left panel). Understanding which step of the anti-cancer immune response is not functional in cancers is crucial to adapt therapies to the cancer phenotype.

## Lack of Tumor Antigens

Tumor antigens can be divided into three main classes: tumor specific antigens (TSA), cancer-germline antigens (CGA), and tumor associated antigens (TAA) (3). TSA are expressed only by cancer cells and not in healthy tissues (4). TSA include mutation-associated neoantigens (MANA) and viral antigens. MANAs arise from DNA mutation/rearrangement in a gene coding sequence and play a crucial role in the recognition of tumor cells by CD8^+^ T cells after immune checkpoint treatment (5). Viral antigens may also represent the target for immune recognition of virus-associated tumors (6). CGA are expressed in tumor cells of different histological origins, but they are silent in normal adult tissues, except in the male germ line and trophoblastic cells (7). Their expression is associated to the demethylation of their promoter. TAA correspond to antigens with low expression in normal tissues and overexpressed in tumor cells, like HER2 (8), or melanocyte differentiation proteins (9).

Tumor mutation burden (TMB) is a quantitative measure of the total number of mutations per coding area of a tumor genome that has been shown to predict responses to ICIs in a range of advanced cancers (10). Tumors with a high TMB are believed to express more MANA. Interestingly, a correlation between MANA and CGA has been observed (11). However, even if some tumors are characterized by low expression of both MANA and CGA, the quantity of tumor neoantigens does not seem to be the main limiting factor for the induction of a T cell response. A recent study by Spranger et al. analyzed the impact of the presence of differentiation antigens, CGA and MANA on T cell infiltration in malignant melanoma. They reported that non-T-cell-inflamed melanomas do not lack antigens for T-cell recognition, arguing for other mechanisms causing the lack of T cell priming and recruitment. Moreover, the number of neoantigens and the mutational load was still comparable between non-T cell-inflamed and T cell-inflamed subtypes in other solid tumors (12). Finally, the kinetics of tumor antigens release, associated with different modalities of spontaneous or induced cancer cell death, may also influence the quality of the T cell response (13).

## Absence of T Cell Priming/Activation

### Defective Recruitment of APCs

The second step of the anti-tumor immune response consists in the presentation of tumor antigens by dendritic cells (DCs), resulting in the priming and activation of specific effector T cells. Several DCs subsets such as classical DCs (type 1 cDC1s and type 2 cDC2s), Langerhans cells, inflammatory DCs and plasmacytoid DCs (pDCs) exist and are specialized in different functions to shape the immune response and cope with the threat of diversity (14). In particular some evidences exist showing that a higher ratio of cDC1s over monocytes/macrophages in the tumor bed favors protective anti-tumoral adaptive immune responses (15). Spranger et al. have also shown in melanoma that MHC expression and DCs infiltration is associated with T cell infiltration (12).

Among DCs, cDC1s excel at inducing anti-tumoral CD8 T cell responses through cross-presentation of exogenous antigens on MHC-I (16–18). A strong correlation between CD8 gene transcript and cDC1s markers was observed, suggesting that lack of T cell activation and infiltration in the non-T-cell-inflamed tumor microenvironment is mainly associated with a defective recruitment and activation of cDC1 (19–21). Moreover, recent papers published by M. Krummel and C. Reis e Sousa teams recently demonstrated a critical role of the cross-talk between cDC1s and NK cells for the CTL infiltration in melanoma (22, 23). In mice, several reports have shown that cDC1s are necessary for the natural rejection of transplanted tumors and for the efficiency of anti-tumoral immunotherapies including ICIs or adoptive transfer of anti-tumoral CD8 T cells (24).

### Lack of T Cell Co-stimulation and Activation After Antigen Presentation

The maturation and activation of antigen-presenting DCs is a critical step for activating an efficient T cell-response. In this context, the DC activation marker DC-LAMP is a good prognostic marker in solid tumors (25). Naive T cells require contact with activated APCs to be primed in an appropriate context of “danger signal” (26). APCs expressing Pattern Recognition Receptors (PRRs) can be directly activated by Pathogen-Associated Molecular Patterns (PAMPs) or Danger Associated Molecular Patterns (DAMPs) to become competent to prime T cell responses (27). Engagement of PRRs on DCs induces NF-κB activation, up-regulation of co-stimulatory molecules, production of cytokines and promotion of cross-priming (28, 29). Various DAMPs are produced by tumor cells undergoing immunological cell death [e.g., calreticulin, HighMobility Group Box 1 protein (HMGB1) or Sin3A Associated Protein 130 (SAP130)] (30). The absence or low production of DAMPs could induce a lack of DCs maturation as well as production of immunosuppressive factors such as transforming growth factor beta (TGF-β) leading to the absence of CD4^+^ T cell help (30, 31). Recent works demonstrate the importance of the protein Formyl Peptide Receptor 1 (FPR1) expressed by tumoral DCs in the anthracycline-induced immunogenic cell death. DCs lacking or presenting a variant of FRP1, failed in antigen presentation and activation of T cells, resulting in poor anticancer immune responses and reduced overall survival in breast and colon cancer (32).

Stimulation of CD40 on APCs through CD40L expressed on helper CD4+ T cells is another crucial step for the activation of APCs to prime CD8^+^ T cells. Moreover, the stimulation of CD40 on DCs regulates the expression of the co-stimulatory molecules CD80 and CD86, enhances the production of cytokines (most notably IL-12 and IFN-I) and promotes the cross-priming to exogenous antigens (33). As a consequence, reduced CD8^+^ T cell responses are largely due to impaired activation of APCs or to the absence of co-stimulation.

## Deficit of Homing to the Tumor Bed

### CD8^+^ T Cell Exclusion by the Immunosuppressive Peritumoral Stroma and Tumor Cell Alterations

When DCs are mature and T-cells correctly primed and activated, the access of T cell to the tumor bed could be compromised by the stromal compartment (34). The exclusion of CD8^+^ T cells from the vicinity of cancer cells was shown to correlate with a poor long-term clinical outcome in colorectal cancer, ovarian cancer and pancreatic ductal adenocarcinoma (35, 36). Interestingly, Spranger et al. reported an inverse relationship between intrinsic β-catenin signaling of tumor cells and intra-tumoral T cells in melanoma. Using a genetically engineered mouse model they showed that melanomas arising from mice with active β-catenin were characterized by an almost complete absence of both CD8+ T cells and cDC1 subsets (37). A second pathway identified to play a role in T cell exclusion is PI3K pathway activation/PTEN loss. Loss of PTEN in tumor cells in preclinical models of melanoma was shown to increase the expression of immunosuppressive cytokines, inhibit T cell-mediated tumor killing and decrease T cell trafficking into tumors. Furthermore, in patients PTEN loss correlated with decreased T cell infiltration at tumor sites and inferior outcome after PD-1 inhibitor therapy (13). PTEN-deficient prostate tumors similarly induce an immunosuppressive tumor microenvironment by upregulating PTPN11/SHP2 and inducing activity of the Jak2-Stat3 pathway (38). Loss of PTEN was recently associated with resistance to anti-PD1 therapy in metastatic uterine leiomyosarcoma (39) and the blockade of this pathway *in vivo* contributed to an improved tumor control (13).

Tauriello et al. investigated how genetic alterations and the tumor microenvironment (TME) interact in a metastatic colorectal carcinoma (CRC) model. A Tumor Growth Factor (TGF)-β activity correlating with T cell exclusion and a low TMB was described (40). Recently, a study associated a TGF-β signature of stromal cells with lack of response to anti PD-L1 in the excluded tumor–immune phenotype (41). Blockade of TGF-β in a pancreatic ductal adenocarcinoma model improved the cure rate of mice by decreasing the presence of immune suppressive cells in the TME and enhancing CD8+ T cell infiltration within the tumor (42).

### Modified Production of Chemokines and Cytokines Affecting Cell Trafficking and Activation

Cytokines and chemokines may influence cell trafficking to the tumor bed. Besides the steady-state influx of immature dendritic cells (iDCs) within tissues, chemokines, abundantly secreted under inflammatory conditions, can provoke influx of iDCs in the tumor bed (43). Lack of those chemokines and the consequent reduced influx of iDCs in the tumor bed can be the cause of the reduced activation and migration of T cells at the tumor site. Chemokines acting on iDCs are the Monocyte Chemoattractant Proteins (CCL2, CCL7, CCL8) as well as CCL3/MIP-1alpha, CCL5/RANTES, and CCL4/MIP-1beta (44). Cytokines are also necessary to generate active DCs: as an example type I interferon (IFN-I) produced by DCs can act in an autocrine manner to generate fully active DC1s (45). Moreover, DC1s are a source of CXCL-9/10 and their absence lead to a reduced production of these chemokines (20). The chemokine CXCL16, produced by DCs, and its receptor CXCR6 for example have been associated with an increased CD4^+^ and CD8^+^ T cell recruitment and a good prognosis in CRC (46). The disruption of the CXCL16/CXCR6 pathway could lead to a reduced tumor T cell infiltration.

The deregulation of trafficking can directly involve T cells: DCs-activated T cells against tumor antigens have to reach the tumor bed to perform their anti-cancer activity. Tumors can disrupt chemokine expression to deregulate the immune response and chemokines involved in effector T-cell recruitment is significantly reduced in tumors lacking a CD8^+^ T-cell infiltrate. CXCL9 and CXCL10 (CXCL11 in humans) are key chemokines in the recruitment of CD8+ T cells engaging the CXCR3 on their surface and their production is generally deregulated in “non-inflamed” tumors (47). CXCL9/10 can be produced by the tumor cell itself where a methylation of chemokine genetic loci results in a reduced CD8^+^ T cell infiltration. The use of demethylating agents restores chemokine production and T-cell recruitment, showing that epigenetic modification is a mechanism of tumor escape which could lead to the lack of immune cells infiltration (48). Tumors can also alter the chemistry of certain chemokines to preferentially recruit myeloid cells: as an example the nitrosylated CCL2 eliminates the ability to recruit CTLs and Th1 effector cells (49), while selectively recruiting myeloid dendritic stem cells (MDSCs) to tumor sites.

## Therapeutic Approaches

**Figure 2:**
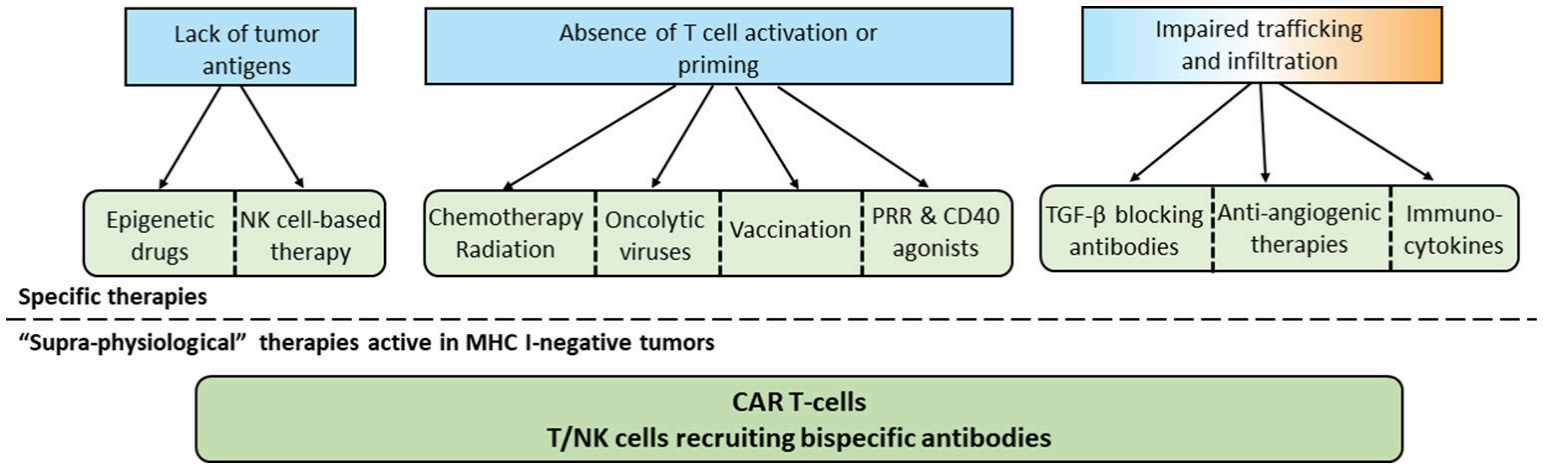
Specific and common approaches to overcome the absence of T cells in tumors. According to the mechanism involved in the lack of T cell infiltration in tumors, specific therapies can be selected. In the case of MHC-I negative tumors or if specific therapies are not sufficient, “supra-physiological therapies” can be used.

Different therapeutic approaches can theoretically be used to overcome the absence of T cell infiltration in tumors. These strategies are summarized in Figure 2. The demonstration that these therapies can effectively transform a cold into hot tumor remains to be done in the clinic in most instances.

### Specific Therapies for Tumors Expressing Few Antigens

#### Demethylating Agents

It has been shown that DNA methyltransferase inhibitors (DNMTi) and histone deacetylase inhibitors can enhance the expression of tumor antigens and components of antigen processing and presenting machinery pathways, as well as other immune related genes (50, 51). These agents can also induce the expression of retroelements such as endogenous retroviruses (ERVs), usually silent and able to induce a type I IFN response (52). Epigenetic drugs have been reported to induce transcription from normally repressed ERV LTR, that may cause ectopic expression of transcripts with canonical or novel open reading frames, leading to the production of immunogenic peptides (53, 54). DNMTi and Histone-lysine N-methyltransferase EZH2 inhibitors have also been shown to reverse epigenetic silencing of Th1-type chemokines in tumor cells, which is negatively associated with CD8^+^ T cells in tumors and patient outcome (48). There is thus a strong rationale to combine epigenetic therapy and immunotherapy and many clinical trials are currently ongoing (55).

#### NK Cell-Based Approaches

Natural killer (NK) cells are lymphocytes of the innate immune system able to recognize and kill tumors lacking self-MHC class I molecules, by recognizing stressed cells. For this reason NK approaches could be suitable in the absence of tumor antigens or in case of deficient antigen presentation machinery (e.g., lack of MHC class I). While a large portion of cancer immunotherapies focus on targeting T cells, NK cell system for therapeutic intervention stays relatively underexplored. Nevertheless, different NK cell-based approaches have been described, such as *ex vivo* activated NK cells or NK cells transduced with a chimeric antigen receptor (CAR) to target specific cancer cell surface antigen (56, 57). Antibodies-mediated targeting of NK activating receptors such as NKG2D and NKP46, or inhibitory receptors such as KIR and NKG2a, is under deep investigation (58). Lirilumab is a fully human antibody directed against KIR2DL-1,-2,-3 inhibitory receptors expressed predominantly on NK cells and is being tested in combination with ipilimumab or nivolumab for the treatment of patients bearing advanced solid malignancies (59).

### Specific Therapies for Tumors With Defective Priming or T Cell Activation

#### Chemo/Radiotherapy Inducing Immunogenic Cell Death (ICD)

Several chemotherapies were found to work mainly in immunocompetent subjects, accumulating evidences that tumor inhibition partially relies on the immune system competence and not only on the direct anti-tumor toxicity of chemotherapy (60). In this regard ICD inducing chemotherapeutic agents can be classified within cancer immunotherapy strategies. Radiotherapy was initially designed to selectively kill tumor cells within the irradiated field. However, emerging evidence indicates that radiotherapy, by inducing ICD, harnesses the host's immune system to attack the tumor cells outside the irradiation field, explaining the “abscopal” effect (regression of tumor lesions outside the irradiation field) (61). Based on this rationale, many trials are ongoing to combine chemotherapy and radiotherapy with PD-1/PD-L1 antibodies. Of note, the same rationale also applies to antibody-drug conjugates (ADCs) with cytotoxic payloads capable of inducing ICD, justifying the initiation of clinical trials combining ADCs and immunotherapy (62).

#### Oncolytic Viruses

Oncolytic viruses are common viruses that can selectively target, replicate in and destroy cancer cells (63). Most oncolytic viruses can induce cancer cell death and directly eliminate tumor cells, but they also initiate systemic immune responses through different mechanisms such as induction of ICD and release of danger signals (DAMPs) and tumor antigens from virus-infected cells. They also release viral PAMPs contributing to APCs maturation that conduct to activation of antigen-specific CD4^+^ and CD8^+^ T cell responses (64). Moreover, the infected cells are directly recognized by the innate immune system such as NK cells or macrophages (65). It has been recently shown in a melanoma phase I trial that use of oncolytic viro-therapy was able to convert a cold into hot tumor, as patients with a low level of immune cell infiltrate and a negative IFNγ signature before treatment responded well to the combination of talimogene laherparepvec with the PD-1 antagonist pembrolizumab (66).

#### PRR Agonists

PRRs consist of five families including Toll-like receptors (TLRs), RIG-I-like receptors (RLRs), nucleotide-binding oligomerization domain (NOD)-like receptors (NLRs), C-type lectin receptors (CLRs), and cytoplasmic DNA sensors. PRRs agonists have notably the ability to activate PRR pathways inducing antigen presentation by myeloid cells residing in the tumor micro-environment (67–69).

TLR agonists showed controversial results in pre-clinical studies by either promoting or inhibiting tumor progression depending on the TLR and the tumor type (70). Multiple TLR agonists are currently in clinical development. As an example, the intra-tumoral injections of a TLR-9 agonist, the CpG-rich oligonucleotide PF-3512676, showed significant activity in both injected and non-injected lesions of B- and T-cell lymphomas when used concomitantly with low-dose (2 × 2 Gy) local irradiation (71).

STING has been shown to play an important role in the innate immune response against cancer. In the TME, tumor cell DNA detected by APCs is correlated with activation of STING pathway that leads to IFN-β production (25), enhancing CD8^+^ T cell priming and trafficking of effector T cells. One STING agonist, ADU-S100 (Aduro Biotech/Novartis), is currently evaluated in phase I clinical trial by intra-tumoral administration in cutaneously accessible tumors (72).

#### CD 40 Agonistic Antibodies

CD40 is broadly expressed on immune cells, predominantly on DCs, B cells and macrophages. A major role of CD40 signaling is to activate and “license” DCs to prime effective cytotoxic CD8^+^ T cell responses. CD40 signaling can also be effectively triggered using agonistic antibodies or CD40L, thus bypassing the need for CD4^+^ helper T cells (73). In preclinical studies, agonistic CD40 antibodies have demonstrated T cell-dependent anti-tumor activity, in particular in combination with conventional chemotherapy and immune checkpoint inhibitors (ICIs). Many CD40 antibodies are under clinical development (74). Toxicity profile is acceptable in monotherapy and combination trials are ongoing (75).

#### Tumor Vaccines

The therapeutic breakthrough provided by ICIs and the demonstration of the role of MANA in T-cell mediated antitumor response have paved the way for a next generation of personalized cancer vaccines based on the use of MANA specific of the tumor. Preclinical results showed induction of efficient antitumor response and clinical trials providing a clinical proof of concept in melanoma have been published (76–78). A specific CD8^+^ and CD4^+^ T cell immune response characterized by the induction or the amplification of a preexisting response against MANA has been shown. Encouraging clinical activity seems to be associated but larger trials are awaited to firmly demonstrate the clinical activity of this therapeutic approach. Theoretically, cancer vaccines may either reinforce the activity and therapeutic margin of ICIs by increasing the number of specific effector T cells, or convert cold into inflamed tumors. The potential limitations are the availability of T cell repertoire in cancer patients and the risk of specific loss of heterozygosity (LOH) of the HLA presenting MANA in advanced metastatic tumors (79).

### Specific Therapies for Tumors With Impaired T Cell Trafficking to the Tumor

#### TGF-β Blocking Antibodies and TGF-β-Receptor Antagonists

TGF-β has been involved in cell proliferation, angiogenesis, epithelial-to-mesenchymal transition, immune infiltration, metastases dissemination, and drug resistance (80). Of note TGF-β produced by the tumor cells mediates alterations in tumor-associated pDCs functions, e.g., impaired capacity to produce IFN type I, leading to a lacking/unbalanced T cell recruitment (81, 82). Recent reports have shown that TGF- β in the peritumoral area was a major factor involved in T cell exclusion from the tumor. Mariathasan et al. used a preclinical model recapitulating T cell exclusion and showed that combination of a TGFβ blocking antibody with a PD-L1 antibody induced T cell penetration into the center of tumors, allowing anti-tumor immunity and tumor regression (41). Several *TGF-*β antibodies or small molecules TGF-β-receptor antagonists are in clinical development and could be tested in this setting.

#### Anti-angiogenic Therapies

The clinical activity of anti-angiogenic drugs is modest when used as single agent. However, it has been shown that anti-angiogenic drugs normalize the tumor vasculature and induce the upregulation of the leukocyte adhesion molecules ICAM-1 and VCAM-1 on tumor endothelial cells (83), leading to increased T cell infiltration (84). These therapies may thus represent a treatment of choice for tumors characterized by T cells blocked in the periphery of the tumor in order to enhance intratumoral penetration of T cells. It has been shown in the clinic that anti-angiogenic therapies could synergize with ICIs in metastatic melanoma (85).

#### Immunocytokines

Cytokines such as IL-2, TNF, IL-12 mediate the influx and expansion of leukocytes at the tumor site. However, these cytokines, and especially IL-12, induce significant toxicity when administrated systemically in clinical trials (86). Next generation immunocytokines combining a fragment from a specific tumor antigen antibody with a modified cytokine are being developed with the aim of activating specifically the immune system inside the tumor to reduce systemic side effects. For example, cergutuzumab amunaleukin (CEA-IL2v), is a novel monomeric CEA-targeted immunocytokine that comprises a single IL-2 variant (IL2v) moiety with abolished CD25 binding (to avoid activation of regulatory T cells) fused to the C-terminus of a high affinity bivalent carcinoembryonic antigen (CEA)-specific antibody devoid of Fc-mediated effector functions. A superior efficacy over the respecting monotherapies was observed with CEA-IL-2v in combination with PD-L1 antibody and ADCC competent antibodies in CEA-positive solid tumor models (87).

### Therapeutic Approaches Active in Different Immune Contexts

#### Adoptive T Cell Therapy and Chimeric Antigen Receptor (CAR) T Cells

Historically, cell-based therapies have focused on cytotoxic T cells targeting MHC-restricted antigens. This approach remains promising, in particular with the development of T cell receptor (TCR)-engineered T cells (88) and improvement of tumor infiltrating lymphocytes (TILs) infusion (89). However, their efficacy may be limited by the tendency of tumors to downregulate MHC molecules. CARs are engineered receptors made of the combination of an antigen binding domain of a monoclonal antibody specific for a cancer antigen (not MHC restricted) together with an intracellular domain of the CD3-zeta chain (90). CAR T cells responses can be further enhanced by addition of costimulatory domains, such as CD28 and CD137 (4-IBB) to support the expansion and persistence of genetically engineered cells *in vivo* (91). The reinfusion of CAR T-cells is preceded by a “lymphodepleting” chemotherapy regimen used to physically create enough space for the expansion and persistence of CAR T cell clones. Over the past decade multiple tumor antigens have been targeted by CARs. Outstanding activity of CAR T cells targeting CD19 has been observed in hematological malignancies, in particular in acute lymphoblastic leukemia and Diffuse Large B cell lymphoma with two CAR T-cell therapies approved by the Food and Drug Administration (FDA) in 2017 (92). The clinical activity of CAR in solid tumors is still to demonstrate and will probably require optimization of CAR functions [for review see (93)].

#### T-Cell Recruiting Bi-specific Antibodies

Bi-specific antibodies (bsAbs) are engineered antibodies that can bind two different antigens. One of the main strategies in the development of bsAbs is the recruitment and activation of immune effector T cells by targeting CD3 domain of the TCR complex (T-cell recruiting bsAbs) together with another antigen abnormally expressed on the tumor cell surface. The approval of catumaxomab (anti-epitelial cell adhesion molecule EpCAM and anti-CD3) and blinatumomab (anti-CD19 and anti-CD3) has become a major milestone in the development of bsAbs. BsAbs can be divided into two categories: immunoglobulin G (IgG)-like molecules and non-IgG-like molecules. Non-IgG-like bsAbs are smaller in size, leading to enhanced tissue penetration, but shorter half-life (94). Currently, more than 60 different bsAbs formats exist, some of them making their way into clinical trials. As for CAR T-cells, activity of T cell-recruiting bsAbs is not dependent on MHC class I expression on tumor cells and both approaches represent thus very promising treatments for MHC I-negative cold tumors. It is however not know whether a minimum threshold of T cells inside the tumor is required for the activity of bsAb, alone or in combination with other therapies. A comparison between CAR T cells and T-cell recruiting bsAbs is proposed in Table 1.

**Table 1:** Comparison between CAR T-cells and T-cell recruiting bi-specific antibodies.

	**CAR T-Cells**	**Bi-specific antibodies**
Mechanism of action	- Direct cancer antigen recognition - Non MHC-restricted - Does not require pre-existing T cell infiltration, independent of receiver T cell characteristics	- Recruitment of immune effector T cells by their CD3 with another antigen expressed on the tumor cell - Non MHC-restricted - May be more dependent on quantity/quality of patients' T cells
Administration	- Single administration - Long half-life (months/years)	- Repeated administration (continuous infusion for non-IgG-like) - Short/intermediate half-life (hours/days)
Tissue penetration	- Homing of the T cells for blood, lymph nodes, and bone marrow	- Non IgG like: enhanced tissue penetration
Toxicity	- Acute reversible neurotoxicity (CD19) - Cytokine release syndrome (CRS)	- Lower toxicity expected - Acute reversible neurotoxicity (CD19) - Cytokine release syndrome (CRS)
Main diseases	- Outstanding activity in some hematological malignancies: B cell acute lymphoblastic leukemia, Diffuse Large B cell lymphoma (CD19) - Clinical trials in solid tumors	- B cell acute lymphoblastic leukemia (CD19) - Clinical trials for many solid tumors including colorectal, ovarian, breast and prostate cancer
Other limitations	- Clinical activity in solid tumors is still to demonstrate - Immunosuppressive microenvironment (rationale for combination with ICIs or use of optimized CAR T-cells) - Target specificity: risk of escape by loss of the target	- Clinical activity in solid tumors is still to demonstrate - Immunosuppressive microenvironment (rationale for combination with ICIs) - Target specificity: risk of escape by loss of the target
Cost and availability	- Long process, manufacturing issues - Cost^+++^	- Immediate availability, less manufacturing and regulatory issues - Cost^+^

## Perspectives

Changing the natural history of a tumor characterized by the absence of T cells remains a great therapeutic challenge. However, as discussed above, many therapeutic approaches can be evaluated in this context. A major issue will be to determine the origin of this lack of T cell response to adapt the therapy to the physiology of the tumor. Figure 2 summarizes the possibilities of treatment according to this tumor context.

Conventional anticancer approaches like chemotherapy and radiotherapy have still a room in the therapeutic armamentarium, not only to potentially induce ICD but also to reduce the tumor burden and thus potentially decrease the selection of immune-resistant clones. In other cases, “*in situ*” vaccination using PRR or CD40 agonists may be used to induce a specific immune antitumor response against naturally presented tumor antigens. Personalized cancer vaccines also represent a very promising strategy especially in highly mutated tumors. Finally, “supra-physiological” approaches like CAR T cells or T-cell recruiting bsAbs could be efficient in tumors characterized by the absence of MHC expression or even LOH of the HLA alleles presenting tumor antigens. It is likely that in the near future, algorithms of treatment will be developed to adapt the therapeutic strategy to the immune context of the tumor, considering also space and time evolution for adequate sequential strategies. At the end, while the efficacy of the therapy in inflamed tumors depends principally in the remove of the breaks induced by the immune activation itself, the conversion of a cold into an inflamed tumor will require a prior combination of therapies to induce immune infiltration and then different immune checkpoint modulators to remove the breaks.

## Author Contributions

PB, TS, VA, JV-G, and SD contributed to the writing. SD and CC contributed to the conception. SV-W, SA, CC, and SD participated in the lecture and corrections.

### Conflict of Interest Statement

SD is also an employee for Cellectis and reports personal fees from AstraZeneca, Elsalys, Erytech Pharma, and Netris Pharma. The remaining authors declare that the research was conducted in the absence of any commercial or financial relationships that could be construed as a potential conflict of interest.
